# Zbtb48 is a regulator of Mtfp1 expression in zebrafish

**DOI:** 10.1038/s42003-025-07666-z

**Published:** 2025-02-22

**Authors:** Sho Yee Carisa Goh, Albert Fradera-Sola, Nadine Wittkopp, Naz Şerifoğlu, Miguel Godinho Ferreira, Rene F. Ketting, Falk Butter

**Affiliations:** 1 Institute of Molecular Biology (IMB), Ackermannweg 4, 55128 Mainz, Germany; 2 Friedrich-Loeffler-Institut, Suedufer 10, 17493 Greifswald, Germany; 3 Institute for Research on Cancer and Aging of Nice (IRCAN), CNRS UMR7284, INSERM U1081, Université Côte d’Azur, 28 avenue de Valombrose, 06107, Nice, CEDEX 2 France

**Keywords:** Ageing, Gene expression

## Abstract

ZBTB48 (also known as TZAP) is a transcription factor that has previously been reported to bind to telomeres and act as a negative regulator of telomere length in human cell lines. To explore whether transcription factor activity and telomere length regulation are conserved at the organismal level in vertebrates, we generate a *zbtb48*^*−/−*^ zebrafish line via CRISPR‒Cas genome editing. The *zbtb48*^*−/−*^ mutants display no obvious physical or behavioral abnormalities in the first two generations. We find no statistically significant changes in telomere length in first-generation adults. However, for the gene regulatory aspect of Zbtb48, similar to that in human cancer cell lines, we observe downregulation of *mtfp1* at both the mRNA and protein levels in the *zbtb48*^*−/−*^ mutants. This suggests that *mtfp1* is an evolutionarily conserved regulatory target of Zbtb48. Further investigation of the spatiotemporal expression of *zbtb48* in previously published zebrafish data reveals low transcript expression in diverse tissues, except in germline stem cells and gametocytes of the gonads. Notably, Mtfp1 protein downregulation is detected in the ovaries of 40 dpf *zbtb48*^*−/−*^ mutants and in the testes of both 40 dpf and 10.5-month-old *zbtb48*^*−/−*^ mutants.

## Abstract (web summary)

The regulation of Mtfp1 by the transcription factor Zbtb48 is evolutionary conserved within vertebrates.

## Introduction

The zebrafish has emerged as an in vivo model for studying telomere regulation because its telomeres share several common properties with those of humans. Similar to those in humans, zebrafish telomeres consist of TTAGGG tandem repeats that typically range from 5 to 15 kilobases in length^1^. Despite the expression of telomerase in somatic tissue, telomeres in zebrafish shorten during aging^2^. In contrast to laboratory inbred mouse strains, zebrafish exhibit telomere deficiency phenotypes already in the first generation of *tert*^*−/−*^ mutants that are correlated with significantly shorter telomeres compared to their wild-type counterparts^1^. The lifespan and telomere length of first-generation *tert*^*−/−*^ mutants are reduced, and they experience premature aging with a rapid decline in fertility^1,3^. Critical short telomeres accumulate primarily in the gut^4^, and tissue-specific rescue of telomerase in the gut suppresses premature aging phenotypes and extends lifespan^5^. Second-generation *tert*^*−/−*^ mutants do not survive to adulthood because the critical short telomeres activate p53-induced apoptosis at the embryonic stage^3^. Consequently, the *tp53*^*M214K*^ mutation in *tert*^*−/−*^ fish also results in partial lifespan extension^6^. In zebrafish, telomerase expression is further required for regeneration of the heart^7^ and for developmental hematopoiesis^8^. Notably, *tert*^*−/−*^ zebrafish mutants exhibit phenotypes comparable to those of dyskeratosis congenita (DC), a bone marrow failure disorder that affects multiple parts of the human body. Overall, these findings make zebrafish a suitable model for studying telomere biology^9^.

In mammals, telomeres are bound by the shelterin complex, which consists of six core proteins (TRF1, TRF2, RAP1, POT1, TIN2, and TPP1), the CST complex (CTC1, STN1, TEN1) and other telomere-associated proteins^10^. These proteins have orthologs in zebrafish, and initial functional characterization has already been conducted.

Loss of Terfa (the TRF2 ortholog) in zebrafish is embryonic lethal, and heterozygous adult fish exhibit accelerated aging^11^. In addition to the induction of DNA damage, *terfa*^*−/−*^ fish experience neurodevelopmental failure due to brain edema during embryo development based on transcriptional misregulation^12^. Generated *pot1*^*−/−*^ and *acd*^*−/−*^ (the TPP1 ortholog) lines die at the larval stage, with a few *acd*^*−/−*^ mutant escapers showing premature aging^13^. Furthermore, *acd* morphants exhibit neural death, heart defects and extensive apoptosis during embryonic development^14^. In contrast, *tinf2*^*−/−*^ (the TIN2 ortholog) and *terf1*^*−/−*^ mutants seemed unaffected and developed normally into adults^13^.

Previously, a phylointeractomic study utilizing telomere pull-down assays coupled with mass spectrometry analysis identified TTAGGG-binding proteins in 16 vertebrate species, including zebrafish^15^. Apart from the core shelterin proteins, several recently characterized direct telomere-binding zinc finger proteins, such as ZBTB10^16^ and ZNF524^17^, bind to the telomeric TTAGGG repeat sequence. One of these zinc finger proteins, ZBTB48 (also known as TZAP or HKR3), showed conserved phylogenetic binding in 13 of the 16 species^15^. ZBTB48 is composed of an N-terminal BTB/POZ domain and a series of eleven adjacent C2H2-type zinc fingers at the C-terminus. Its human ortholog directly interacts with the TTAGGG telomere repeats via its eleventh zinc finger and adjacent C-terminal arm^18^. Telomere sequence recognition is exerted by a recurring RxxHxxR motif in the telomeric zinc finger proteins ZBTB10, ZNF524 and ZBTB48^17,19^.

Knockout of ZBTB48 in telomerase-positive human HeLa cancer cells and mouse embryonic stem cells results in telomere elongation^20,21^. Conversely, the overexpression of ZBTB48 in human U2OS cancer cells maintaining telomere length via the alternative lengthening of telomere (ALT) pathway results in telomere shortening^21^. This alteration was accompanied by the accumulation of extrachromosomal telomere repeat (ECTR) DNA; thus, it was proposed that ZBTB48 is involved in telomere trimming^21^. Further investigation revealed that ZBTB48 binds to telomeres in an open chromatin state induced by the absence of the ATRX/DAXX protein complex^22^. Although ZBTB48 has been extensively studied in cancer cell lines, alterations in this gene have been found in only 5% of tumors, and the prognosis varies depending on the tumor type^23^.

Apart from its telomeric functions, ZBTB48 can also regulate genes via direct binding to promoter regions of a selected subset of genes where it acts as a transcription activator. In human cancer cells, ZBTB48 knockout strongly downregulated mitochondrial fission process 1 (MTFP1), mirroring the mitochondrial matrix reorganization phenotype of MTFP1 depletion^20^. ZBTB48 was recently reported to regulate B-cell-specific CIITA expression in mice by acting as a pioneer transcription factor^24^.

To gain further insight into the telomeric and gene regulatory functions of ZBTB48 in an in vivo animal model, we established a *zbtb48*^*−/−*^ zebrafish line.

## Results

### Identification of telomeric proteins in zebrafish by quantitative mass spectrometry

**Fig. 1:**
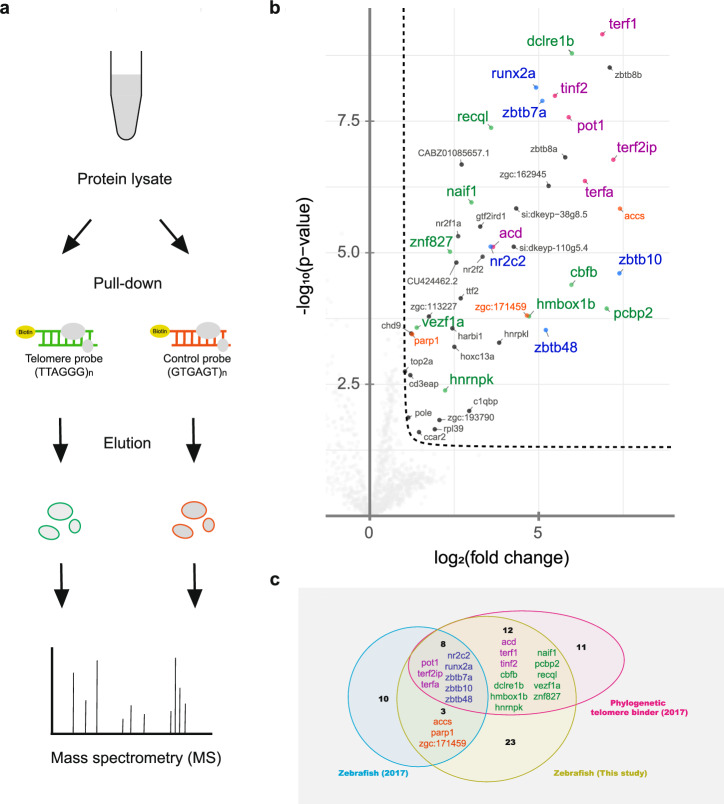
Telomere pull-down with the zebrafish cell line. **a** Schematic diagram showing an overview of the telomere pull-down experiment using nuclear lysate from the zebrafish fin fibroblast BRF41 cell line. **b** Volcano plot visualizing the results obtained by label-free quantification of the bound proteins. The pull-down experiment was conducted in quadruplicate using either concatenated telomeric TTAGGG or a control scrambled GTGAGT oligonucleotide as bait. The results were log-transformed and plotted on the x-axis as log_2_(fold change) and on the y-axis as -log_10_(*p*-value). The protein enrichment threshold was set at a fold change >2 and a *p*-value < 0.05 (Welch’s t test) with c = 0.05. Enriched proteins were annotated with their gene names and color-coded: shelterin subunits (magenta), proteins that were previously found in the study by Kappei et al. to be enriched in zebrafish (orange), and telomere-binding proteins previously identified as phylogenetically conserved in other species (green), which were also enriched in zebrafish (blue). **c** Comparison of the previous telomere zebrafish pull-down, the phylogenetic conserved binders and the candidates from the current study.

To recapitulate and expand on the previously reported putative telomere-binding proteins in zebrafish, we combined a telomere pull-down assay with label-free quantitative mass spectrometry (Fig. 1a)^15^. To this end, we concatenated chemically synthesized DNA oligonucleotides containing the telomeric TTAGGG sequence or a shuffled control sequence by in vitro ligation. The concatenated telomeric and control oligonucleotides were biotinylated in vitro, immobilized on paramagnetic streptavidin beads and incubated in quadruplicate with nuclear lysate from the zebrafish fin fibroblast BRF41 cell line. After several washes, the bound proteins were eluted from the beads, digested into tryptic peptides, and measured using a high-resolution mass spectrometer with subsequent label-free quantitative analysis based on MaxLFQ. In this experiment, we quantified 1,269 proteins, 46 of which were more than twofold enriched, with a *p*-value < 0.05 for the telomeric oligonucleotide (Fig. 1b; Supplementary Data 1). Among these proteins were all six orthologs of the core shelterin complex: Terfa, Terf1, Pot1, Tinf2, Terf2ip (also known as Rap1), and Acd (also known as Tpp1). By comparing our results to a previous screen covering 16 different vertebrates^15^, we successfully enriched all eight previously reported zebrafish candidates conserved among at least 5 species: the shelterin subunits Pot1, Terf2ip and Terfa, as well as the nuclear receptor Nr2c2, the transcription factor Runx2a, the zinc finger protein Zbtb7a, and the direct telomere-binding proteins Zbtb10 and Zbtb48 (Fig. 1c). Additionally, we also found Accs and Parp1, and zebrafish protein Zgc:171459 identified in the previous zebrafish experiment^15^. However, our current screen generated a more comprehensive list of candidates for zebrafish, as we listed here 9 additional proteins whose orthologs were already identified as TTAGGG binders in at least 5 other species in the previous phylointeractomics screen. These proteins include the Cbfb subunit of the heterodimeric Runx/Cbfβ complex, the shelterin-associated protein Dclre1b (also known as the 5’ exonuclease Apollo), the two direct telomere-binding proteins Hmbox1b and Znf827, the poly(C)-binding proteins Hnrnpk and Pcbp2, the nuclear apoptosis-inducing factor Naif1, the DNA helicase Recql, and the zinc-finger protein Vezf1a. Furthermore, we also identified two other proteins (i.e., Zbtb8a and its paralog Zbtb8b) that were more than 30-fold enriched and have not been reported in the previous study. Overall, of our 46 TTAGGG binders found in zebrafish, 20 overlap with the identified phylogenetic conserved candidates in the previous phylointeractomics study and thus constitute a solidified list of known and possible telomere-binding proteins.

### Generation of a *zbtb48* knockout zebrafish line via CRISPR‒Cas

**Fig. 2:**
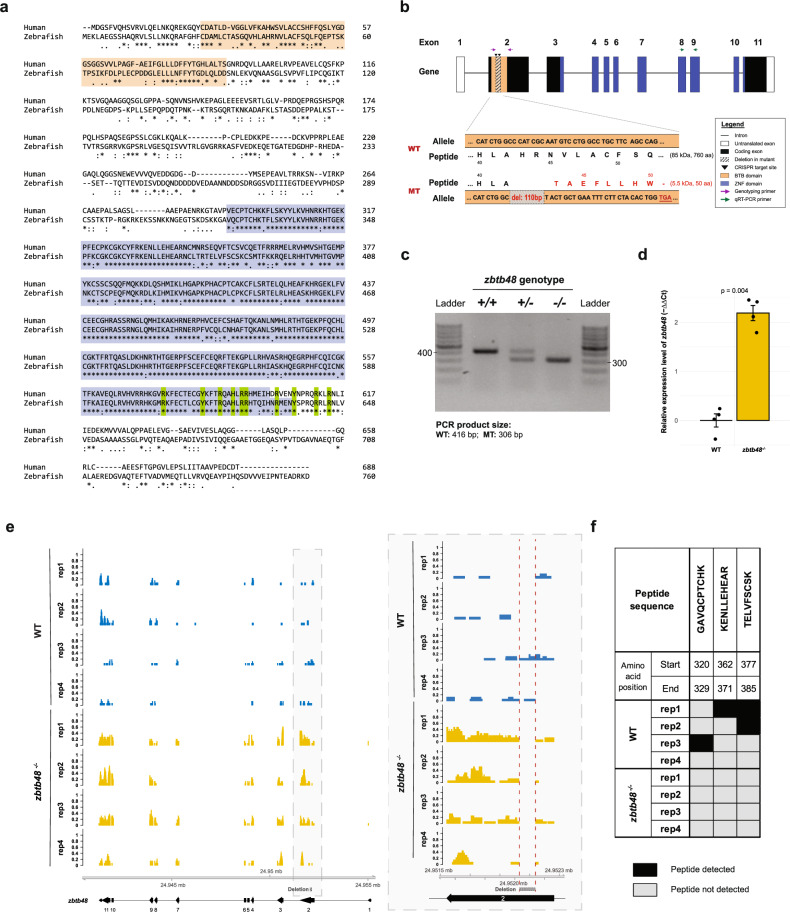
Generation and validation of the *zbtb48* CRISPR‒Cas knockout zebrafish line. **a** Protein sequence alignment of human ZBTB48 and zebrafish Zbtb48 showing greater homology in the telomere-binding zinc finger domain (80% identity; blue) than in the BTB domain (42% identity; beige). The ten amino acids highlighted in green that are involved in telomere binding activity in humans (Zhao et al.) are conserved in zebrafish. **b** The zebrafish *zbtb48* gene consists of 11 exons (not drawn to scale). To create the *zbtb48* CRISPR‒Cas knockout zebrafish line, a pair of guide RNAs was designed to target the second exon, resulting in a 110-base pair frameshift deletion with a premature stop codon. **c** Results of the genotyping of *zbtb48* CRISPR‒Cas knockout zebrafish using agarose gel electrophoresis. **d** Comparison of *zbtb48* mRNA levels between *zbtb48*^*−/−*^ mutants and their wild-type counterparts via qRT‒PCR of 5 dpf larvae. The experiment was conducted in biological quadruplicate (25 pooled larvae), each with technical triplicates. Error bars represent the standard error of the mean (SEM), and *p*-values were calculated using two-tailed Welch’s t test. **e** mRNA sequencing tracks of the *zbtb48* gene region in 5 dpf larvae of wild-type and *zbtb48*^*−/−*^ mutant larvae. A magnified image of the boxed area is shown on the right. The CRISPR deletion is marked below the track. **f** Table showing the Zbtb48 peptides detected via mass spectrometry analysis of a telomere pulldown with lysates extracted from 5 dpf wild-type and *zbtb48*^*−/−*^ larvae.

Among our highly enriched candidates was Zbtb48, which has recently been characterized as a direct telomere-binding protein in human HeLa and U2OS cells^20,21^. The zebrafish ortholog also features an N-terminal BTB domain and eleven C2H2-type zinc fingers (ZNFs) at the C-terminus (Fig. 2a). The amino acid sequence conservation of the BTB domain (aa 28–aa 93; 42% identity) is lower than that of the zinc finger region (aa 322–aa 637; 80% identity). Notably, the 10 amino acid residues involved in telomere binding in the region of zinc finger 11 and the adjacent C-terminal region are identical between human and zebrafish Zbtb48^18^.

To investigate the role of Zbtb48 in zebrafish, we utilized a CRISPR-Cas9 system to generate a *zbtb48* knockout zebrafish line. Using a pair of guide RNAs targeting the *zbtb48* gene, we successfully deleted 110 base pairs from the second exon (Fig. 2b) and confirmed the deletion by PCR (Fig. 2c). We found a significant 4.6-fold increase (*p* = 0.004) in *zbtb48* mRNA expression in the *zbtb48*^*−/−*^ mutants sampled at 5 days post fertilization (dpf) (Fig. 2d). This observation was mirrored in the RNA-seq data of 5 dpf larvae (Fig. 2e), where the mRNA level increased 3.2-fold (*p* = 6.59e^−08^). Further examination of the deletion site in the RNA-seq tracks revealed a lack of sequencing reads in this region for the *zbtb48*^*−/−*^ mutants (Fig. 2e, right). The deletion induced a frameshift mutation resulting in a 50 amino acid (5.5 kDa) long Zbtb48 mutant protein that lacked the ZNF domain essential for telomere binding. To confirm the loss of telomere binding of this truncated Zbtb48 protein, we performed a telomere pull-down assay coupled to mass spectrometric analysis with protein lysates from 5 dpf wild-type and *zbtb48*^*−/−*^ larvae. Zbtb48 peptides were detected in the pull-down with lysates from the wild-type larvae but not in the pull-down with lysates from the *zbtb48*^*−/−*^ larvae despite the heightened *zbtb48* mRNA expression level (Fig. 2f). While no shelterin subunits were among the enriched proteins, Nr2f2 identified from the telomere pull-down with BRF41 lysate (Fig. 1b), was significantly enriched by 4.4-fold (*p* = 0.005) in *zbtb48*^*−/−*^ mutants. (Supplementary Fig. 1 and Supplementary Data 1). In summary, we generated a zebrafish line with a telomere-binding defunct Zbtb48.

### Breeding and investigation of telomere length in *zbtb48*^*−/−*^ mutants

**Fig. 3:**
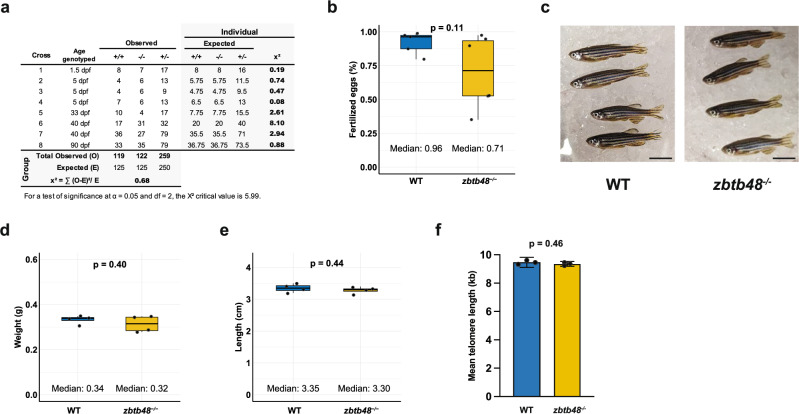
*zbtb48*^*−/−*^ fish have no apparent phenotype. **a** Table showing the chi-square test results for 500 offspring from eight crosses between *zbtb48*^*+/−*^ parents to determine the Mendelian genotype distribution. The test was performed on individual crosses (right) and overall as a group (bottom). For a test of significance at α = 0.05 with 2 degrees of freedom (df), the critical value of the chi-square (X²) is 5.99. **b** Boxplot showing the percentage of fertilized eggs from second-generation *zbtb48*^*−/−*^ mutant males and their wild-type counterparts, both at 1.5-year-old, crossed with wild-type females. Each dot represents a mating setup (*n* = 6 for *zbtb48*^*−/−*^ mutants and *n* = 5 for wild-type), indicating the percentage of fertilized eggs relative to the total number laid. *P*-values were calculated using Welch’s t-test. **c** Photograph of second-generation *zbtb48*^*−/−*^ male zebrafish and their wild-type counterparts at 10.5 months of age. The scale bar equals 1 cm. Boxplot for weight (**d**) and size (**e**). Measurements were performed on four specimens, and *p*-values were calculated by Welch’s t test. **f** Bar plot of the mean telomere length of wild-type and *zbtb48*^*−/−*^ mutant fish (*n* = 3 per genotype) quantified via telomere restriction fragment (TRF) analysis of the caudal fin collected from the first generation of 8-month-old males. Error bars represent the standard error of the mean (SEM), and *p*-value was determined by an unpaired two-tailed t test (Mann–Whitney).

The knockout of some telomere-binding proteins was associated with a reduction in fitness or lethality in offspring. To examine the genotype distribution of our offspring bred from *zbtb48*^*+/−*^ parents, 500 offspring from eight crosses were genotyped at various developmental stages. Generally, the distribution of all crosses followed a Mendelian inheritance pattern, with the exception of cross 6 (Fig. 3a). The results also suggest no significant reduction in the fitness of the *zbtb48*^*−/−*^ mutant during development. Our first inbred generation of *zbtb48*^*−/−*^ mutant fish was alive for two years before the study concluded. Unlike in *tert*^*−/−*^ mutants^1,4^, we did not observe any obvious difference in lifespan between the *zbtb48*^*−/−*^ mutants and their wild-type counterparts. Additionally, the *zbtb48*^*−/−*^ mutants were fertile, and we successfully bred a second generation of *zbtb48*^*−/−*^ mutants through in-crossing. This second generation of *zbtb48*^*−/−*^ mutants was also viable and fertile even at an older age. A fertility test was performed on six *zbtb48*^*−/−*^ mutant males and five wild-type fish at 1.5 years by crossing with wild-type females in a single mating. Despite the second-generation *zbtb48*^*−/−*^ mutants (71%) showed a lower median of fertilized eggs as compared to their wild-type counterparts (96%), the difference was not statistically significant (*p* = 0.11) (Fig. 3b). Neither in the first nor second generation did we observe any physical or behavioral abnormalities in *zbtb48*^*−/−*^ mutants. For example, we compared the appearance (Fig. 3c), weight (Fig. 3d), and length (Fig. 3e) of 10.5-month-old males of second-generation *zbtb48*^*−/−*^ mutants and their wild-type specimens.

Next, we quantified the telomere length of 8-month-old wild-type and *zbtb48*^*−/−*^ males from the first incross by performing telomere restriction fragment (TRF) analysis on the collected caudal fins. We detected no change in the mean telomere length of the *zbtb48*^*−/−*^ fins compared to that of the wild-type controls (p = 0.46) (Fig. 3f; Supplementary Fig. 2). As telomere length homeostasis was not affected in *zbtb48*^*−/−*^ fish, we focused on the transcriptional activity of Zbtb48.

### Transcriptomic and proteomic profiling

**Fig. 4:**
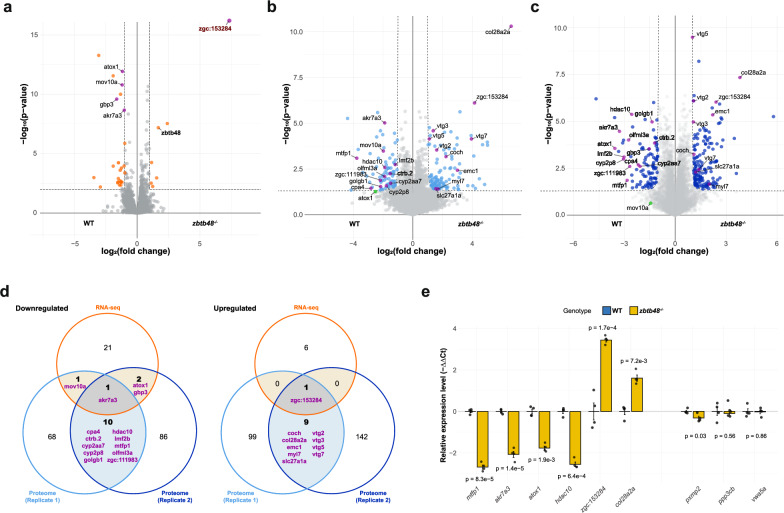
Transcriptomic and proteomic profiling of *zbtb48*^*−/−*^ mutants at 5 dpf. **a** Volcano plot with quantitative comparison of RNA sequencing (RNA-seq) data from 5 dpf wild-type and *zbtb48*^*−/−*^ mutant larvae (*n* = 4). The results were log-transformed and plotted on the x-axis as log_2_(fold change) and on the y-axis as -log_10_(*p*-value) (Welch’s t test). The transcript enrichment thresholds were set at a fold change > |2| and a *p*-value < 0.01. Genes annotated in this plot were also differentially expressed in the proteome analysis (**b** and **c**). Note: the *p*-value (4.14e^−44^) of *zgc:153284* is beyond the upper limit of the y-axis. **b**, **c** Volcano plots for two biologically independent proteome analyses conducted on pooled 5 dpf wild-type and *zbtb48*^*−/−*^ mutant larvae. The results were log-transformed and plotted on the x-axis as log_2_(fold change) and on the y-axis as -log_10_(*p*-value) (Welch’s t test). The protein enrichment threshold was set at a fold change > |2| and a *p*-value < 0.05. **d** Venn diagrams showing downregulated (left) and upregulated (right) transcripts or proteins in the *zbtb48*^*−/−*^ mutants compared to the wild-type. **e** qRT‒PCR analysis of selected genes in 5 dpf larvae of wild-type and *zbtb48*^*−/−*^ mutant zebrafish. The set includes differentially expressed genes from this study (left) and previously reported human target genes (Jahn et al.) (right). The experiment was conducted in biological quadruplicate (25 pooled larvae) with technical triplicates each. Error bars represent the standard error of the mean (SEM), and *p*-values were calculated using two-tailed Welch’s t test.

In addition to controlling telomere length, Zbtb48 has been reported to be a transcriptional regulator in human cells^20^. To investigate whether this function is conserved in zebrafish, we conducted transcriptome (Fig. 4a) and proteome (Fig. 4b, c) analyses of 5 dpf *zbtb48*^*−/−*^ mutant and wild-type larvae.

For RNA sequencing (RNA-seq) analysis (Fig. 4a; Supplementary Data 2), we collected four wild-type and *zbtb48*^*−/−*^ siblings bred from *zbtb48*^*+/−*^ parents through single mating as biological replicates. Among the 32,058 genes quantified by RNA-seq, only 25 genes exhibited downregulation, and 7 genes showed upregulation in the mutant (fold change > |2|, *p*-value < 0.01). In agreement with our qPCR data (Fig. 2d), among these upregulated genes was also *zbtb48*. Notably, *zgc:153284*, which is predicted to be involved in cell redox homeostasis, was the most strongly deregulated gene, with a 163-fold (*p* = 4.14e^−44^) increase in expression in the *zbtb48*^*−/−*^ larvae.

We conducted the proteome experiment in two biological replicates with independently collected samples. Wild-type and *zbtb48*^*−/−*^ larvae were bred from parents of the respective genotype, and they were pooled from a single mating. In the first replicate (Fig. 4b; Supplementary Data 3), a total of 8,075 proteins were quantified, with 80 downregulated and 109 upregulated (fold change > |2|, *p*-value < 0.05). In the second experiment from a new crossing (Fig. 4c; Supplementary Data 3), a total of 7601 proteins were quantified, 99 of which were downregulated and 152 of which were upregulated. Between the two replicates, we observed some variation, and only 11 proteins (Akr7a3, Cpa4, Ctrb.2, Cyp2aa7, Cyp2p8, Golgb1, Hdac10, Lmf2b, Mtfp1, Olfml3a and Zgc:111983) were downregulated, while 10 proteins (Coch, Col28a2a, Emc1, Myl7, Slc27a1a, Vtg2, Vtg3, Vtg5, Vtg7 and Zgc:153284) were upregulated in *zbtb48*^*−/−*^ zebrafish according to both proteome analyses (Fig. 4d). To gain deeper insights into the dysregulated genes, gene ontology analysis of biological processes (GOBP) was performed on both transcriptomic and proteomic data. Although no significant downregulated GOBP terms were identified in the transcriptomic data, biological processes related to transition metal ion transport were consistently and significantly downregulated in both proteomic datasets. In contrast, the upregulated biological processes exhibited differences between the two gene regulatory layers. The transcriptomic data showed a broad range of processes, including translation and nucleotide metabolism. Meanwhile, the first proteomic replicate primarily highlighted processes related to proteolysis and apoptosis, while the second proteomic replicate featured macromolecule and transcription-related processes (Supplementary Fig. 3).

While no clear trend in dysregulated GOBP terms was observed, a small set of genes consistently exhibited significant changes in transcript and protein expression levels (Fig. 4d). This includes *akr7a3, atox1, gbp3* and *mov10a*, which were downregulated, while *zgc:153284* was upregulated. We validated the transcript downregulation of *akr7a3* and *atox1*, as well as the upregulation of *zgc:153284* at 5 dpf by qRT‒PCR (Fig. 4e). Additionally, consistent with the proteome data, we detected downregulation of the *hdac10* and *mtfp1* transcripts and upregulation of the *col28a2a* transcript in *zbtb48*^*−/−*^ larvae, which were not significantly different at the transcript level according to the chosen RNA-seq cut-offs (Fig. 4e). These genes cover diverse biological aspects (Supplementary Data 3), but one of them, Mtfp1, is particularly interesting because its downregulation has also been reported in human ZBTB48 knockout cell lines^20^. We thus extended our investigation to other genes reported to be regulated by ZBTB48 in humans, namely, *PXMP2*, *PP3CB*, and *VWA5A*, by examining the transcription levels of their zebrafish orthologs via qRT‒PCR. There were no differences in the expression of *pp3cb* or *vwa5a* between *zbtb48*^*−/−*^ and wild-type zebrafish larvae at 5 dpf, and only a slight 1.2-fold (*p* = 0.03) decrease in *pxmp2* expression was detected (Fig. 4e). Therefore, strong *mtfp1* downregulation upon *zbtb48* knockout was the most pronounced similarity at the transcript and proteome levels between humans and zebrafish.

### Spatiotemporal expression of *zbtb48*

Based on our proteome and RNA-seq data, Zbtb48 protein expression was low at 5 dpf because it was not detected in the proteome measurements and had a low number of transcript reads. Therefore, we assessed Zbtb48 expression based on publicly available data.

**Fig. 5:**
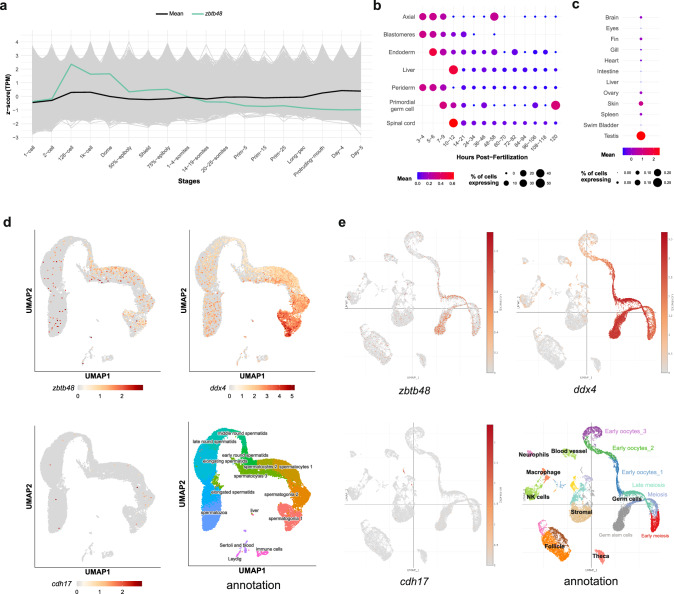
Spatiotemporal expression of *zbtb48* transcripts in larvae and adults. **a** Developmental expression profile with *zbtb48* mRNA highlighted (green thick line) in whole larvae replotted from previously published data (White et al.). Heatmap of *zbtb48* expression in different tissues during the first five days of development (**b**) based on data from Farrell et al. and Sur et al. or in adult tissues (**c**) based on data from Jiang et al. **d** UMAP plots of single-cell RNA sequencing (scRNA-seq) of testes from 5- to 22-month-old fish. Figures were downloaded from the public website (Sposato et al.). **e** UMAP plots of scRNA-seq data from ovaries of 40 dpf fish. Figures were downloaded from a public website (Liu et al.). For **d** and (**e**), the top left panel shows the *zbtb48* mRNA expression level, the top right panel shows *ddx4* (also known as *vasa*, which is highly expressed in germ cells), the bottom left panel shows *cdh17* (not expressed in germ cells), and the bottom right panel shows the cell population annotation.

Using a developmental zebrafish study^25^, we detected high *zbtb48* transcript levels in early development prior to zygotic genome activation, suggesting that *zbtb48* mRNA is maternally loaded into the embryo (Fig. 5a). Transcript levels gradually decrease during development, and at 10–12 hpf, the liver and spinal cord show the highest *zbtb48* transcription levels, followed by primordial germ cells (PGCs) at half of the level^26,27^ (Fig. 5b). By 5 dpf (or 120 hpf), the *zbtb48* transcript became predominantly expressed in PGCs. Due to the strong expression of *zbtb48* transcripts in larval PGCs, we anticipated similar high expression in adult gonads. Therefore, we extended our investigation with a previously published RNA-seq dataset of adult tissues^28^ and found prominent expression in the testis but 30-fold lower expression in the ovary (Fig. 5c). In addition to those in the gonads, low levels of the *zbtb48* transcript were also detected in the skin, brain, fins and spleen.

We speculated that the *zbtb48* transcript might be more highly expressed in highly proliferative tissue due to its involvement in telomere regulation. A single-cell RNA sequencing (scRNA-seq) study of zebrafish testes^29^ revealed the highest *zbtb48* expression in spermatocytes (undergoing meiosis), followed by spermatogonia (stem cells) and then early round spermatids, irrespective of age (Fig. 5d). Notably, the expression of *zbtb48* is high but restricted to a low number of cells, in contrast to *cdh17*, a gene expressed in the nephrons, liver and intestine^30^ that serves as a negative control, and *ddx4* (also known as *vasa*), which is strongly expressed in the germline^31,32^. Consistently, published scRNA-seq data of 40 dpf old juvenile ovaries^33^ also revealed the highest *zbtb48* expression in the germline stem cell population, atop meiotic cells and early oocytes I, compared to *cdh17* as a negative control and *ddx4* as a positive control (Fig. 5e). Overall, the data supported the expression of *zbtb48* transcripts in the gonads, albeit at a small subset of cells and at lower levels than those of the germ cell marker *ddx4*.

We thus decided to focus our investigation on the juvenile gonads of 40 dpf fish. To easily distinguish the gonads, we crossed our *zbtb48*^*−/−*^ zebrafish with a transgenic line expressing eGFP under the *ddx4* promoter, also called *vasa* (*Tg(vasa: eGFP*))^34^. For this study, we used offspring from a single mating of *Tg(vasa:eGFP);zbtb48*^*+/−*^ and collected ovaries from females and testes from male offspring for proteome analysis.

**Fig. 6:**
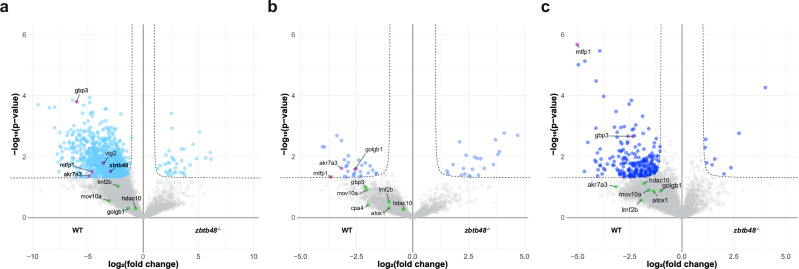
Proteomic analysis of *zbtb48*^*−/−*^ gonads. Volcano plots of the proteome analysis of ovaries from 40 dpf (**a**), testes from 40 dpf (**b**) and testes from 10.5-month-old (**c**) wild-type and *zbtb48*^*−/−*^ mutant fish. Biological replicates of 40 dpf ovaries (*n* = 3) and 40 dpf and 10.5-month-old testes (*n* = 4) were measured for each genotype. The results were log-transformed and plotted on the x-axis as log_2_(fold change) and on the y-axis as -log_10_(*p*-value) (Welch’s t test). The protein enrichment thresholds were set at a fold change > |2| and a *p*-value < 0.05. Proteins common to Fig. 4d were annotated.

Among the 6015 proteins quantified via proteome analysis of the ovaries of *zbtb48*^*−/−*^ versus wild-type zebrafish, 850 proteins were downregulated, and 38 proteins were upregulated in *zbtb48*^*−/−*^ mutants (Fig. 6a; Supplementary Data 4). In contrast, using the same threshold (fold change > |2|, *p*-value < 0.05) among 5891 quantified proteins in the testis experiment, only 30 proteins were downregulated, and 22 proteins were upregulated in *zbtb48*^*−/−*^ mutants (Fig. 6b; Supplementary Data 4). Notably, there was a stark difference in the number of downregulated proteins between the ovary and testis in 40 dpf zebrafish. Among the 11 proteins downregulated in the 5 dpf *zbtb48*^*−/−*^ larvae, Golgb1 was also significantly downregulated in the testis, as were Akr7a3 and Mtfp1 in both the ovary and testis.

To better recapitulate the previously published scRNA-seq data for mature adult testes at 5–22 months of age^29^, we conducted another proteome analysis with 10.5-month-old second-generation *zbtb48*^*−/−*^ mutants. Of the 5419 proteins quantified, 227 were downregulated and 9 were upregulated in the *zbtb48*^*−/−*^ mutants. Thus, the number of downregulated proteins increased with age. Notably, among the downregulated proteins we could recapitulate Mtfp1 from previous 40 dpf ovary and testis experiments, with Mtfp1 showing the strongest downregulation among all measured proteins in 10.5-month-old testes (Fig. 6c; Supplementary Data 4). While *mtfp1* was also downregulated at transcript level in the testes of 1-year-old first-generation *zbtb48*^*−/−*^ fish (Supplementary Fig. 4a), unlike for the 5 dpf embryos, no significant increase in *zbtb48* transcript levels was detected.

GOBP analysis of these proteome datasets did not reveal any consistent biological processes. Unlike the downregulation of transition metal ion transport at 5 dpf, the ovaries and testes of *zbtb48*^*−/−*^ mutants at 40 dpf showed downregulation mainly related to carbohydrate metabolism, with no significant downregulation in the testes at 10.5 months. Instead, the 10.5-month-old testes exhibited upregulation of transcription-related processes, while no significant upregulation was observed in either gonad at 40 dpf (Supplementary Fig. 4b).

## Discussion

We performed a telomere interactomics screen with lysates from a zebrafish cell line and extended the list of previously identified known and putative telomeric proteins. Among the 46 proteins that we found to be enriched with the TTAGGG oligonucleotide, 20 were already shown to recognize TTAGGG repeats in other vertebrate species^15^. These proteins include members of the shelterin complex and other previously reported direct telomere binding proteins, such as Hmbox1^35^, Zbtb10^16^, Znf827^36^, and Zbtb48^20,21^. We decided to focus on Zbtb48, which has previously been reported to be a negative regulator of telomere length in human cancer cell lines and mouse stem cells^20,21^, by generating a *zbtb48* knockout zebrafish line via CRISPR technology. However, we did not observe a telomere lengthening phenotype in our first-generation *zbtb48*^*−/−*^ mutants. Thus, at present, we cannot comment on whether *zbtb48*^*−/−*^ zebrafish require additional generations to exhibit telomere lengthening or whether Zbtb48 is not involved in telomere homeostasis in zebrafish. Additionally, there may be other factors that act redundantly with Zbtb48 to establish the telomere length mean in zebrafish. Therefore, a further long-term study of *zbtb48*^*−/−*^ zebrafish mutants will be required to address this question.

Apart from its role as a telomere length regulator, ZBTB48 was also reported to be a transcription activator for a small group of genes^20^. Similarly, we observed gene regulatory changes in *zbtb48*^*−/−*^ zebrafish in the first and second generations. In this study, we measured the transcriptome and proteome of 5 dpf zebrafish larvae as well as the proteome of ovaries from 40 dpf and testes from 40 dpf and 10.5-month-old zebrafish. Similar to the human ZBTB48 studies, in the *zbtb48*^*−/−*^ mutants, we observed more downregulated genes (*n* = 25) than upregulated genes (*n* = 7) in our 5 dpf transcriptome, which is close to the low number of downregulated genes reported in U2OS (*n* = 23) and HeLa (*n* = 11) cells. Although this trend was not observed in the proteomic data collected at 5 dpf, a skewed distribution later manifested in the proteome of the gonads, with 40 dpf ovaries (850 downregulated, 38 upregulated) and 10.5-month-old testes (227 downregulated, 9 upregulated) showing a stronger trend than 40 dpf testes (30 downregulated, 22 upregulated).

The analyses revealed a small consistent group of genes that were repeatedly downregulated. One of the candidates is the nuclear-encoded mitochondrial protein *mtfp1*, which was previously reported to be downregulated in human ZBTB48 knockout cancer cell lines^20^. Its downregulation was detected via qRT‒PCR and proteomic analysis of 5 dpf *zbtb48*^*−/−*^ larvae and adult testes, as well as in the proteomes of the juvenile ovaries and testes. Apart from *mtfp1*, we also found other candidates that were previously not reported in human cell lines. These genes are aldo-keto reductase *akr7a3* and the guanylate binding protein *gbp3* (involved in the innate immune response), which were consistently downregulated in the transcriptome and most of our proteome analyses. Conversely, Zbtb48 knockout increased the transcript levels of *zgc:153284*, which is involved in cell redox homeostasis, and consequently, protein upregulation was observed in the proteome at 5 dpf. Unlike the previously mentioned candidates, its upregulation was not detected in the gonad proteomes.

We also found that Zbtb48 is generally expressed at low levels in tissues because it was barely detected in our proteomes except for 40 dpf ovaries. The scRNA-seq expression data from various sources^29,33^ show that the *zbtb48* transcript is expressed primarily in the stem cells and gametocytes of the gonads. Since germline stem cells are constantly dividing and rely on telomerase to replenish their telomere length to support ongoing cell division for reproduction, it is conceivable that high *zbtb48* expression helps to reinforce the upper limit of telomere length.

Overall, we established a *zbtb48*^*−/−*^ zebrafish line that can be used as an organismal model to further study the function of *zbtb48* in gene regulation and telomere biology. Although the study of telomere homeostasis in this animal model requires further long-term investigations, we successfully recapitulated the previously reported *zbtb48*-*mtfp1* regulatory loop in zebrafish. The finding that Mtfp1 is an evolutionarily conserved target of Zbtb48 may suggest a relationship between the role of Zbtb48 in telomere maintenance and mitochondrial regulation^37–39^. However, further investigations are needed to elucidate the function of Zbtb48 in zebrafish.

## Methods

### Zebrafish cell culture

The zebrafish-derived fibroblast line BRF41 (CVCL_4131) was cultured in Leibovitz’s L-15 medium (Gibco) supplemented with 15% fetal bovine serum (Gibco) and 1% penicillin‒streptomycin (Sigma). The cells were maintained at 33 °C in an incubator and split 1:5 by trypsinization every three to four days for passaging.

### Preparing protein extracts from BRF41 cells

Nuclear protein extracts were prepared from BRF41 cells as previously described^15^. The cells were incubated in hypotonic buffer (10 mM HEPES (pH 7.9), 1.5 mM MgCl_2_, and 10 mM KCl) on ice for 10 min and then supplemented with 0.2% Igepal CA630 (Sigma) and protease inhibitors before they were homogenized with 50 strokes with a douncer. Nuclei were pelleted and washed with 1X PBS. Nuclear proteins were extracted from the nuclei by incubating them in hypertonic buffer (420 mM NaCl, 20 mM HEPES (pH 7.9), 20% glycerol, 2 mM MgCl_2_, 0.2 mM EDTA, 0.1% Igepal CA630 (Sigma), 0.5 mM DTT and protease inhibitors) for 1 h at 4 °C on a rotating wheel. After centrifugation at maximum speed for 1 h at 4 °C, the supernatant containing the nuclear protein extract was collected. The protein concentrations were measured using a Bradford assay (Bio-Rad).

### Telomere pull-down

Telomere pull-down was performed as described^15^. Briefly, oligonucleotide baits were biotinylated and immobilized on paramagnetic streptavidin beads (Dynabeads MyOne C1, Thermo Scientific). For each replicate, 450 µg of the beads were incubated with 400 µg of protein lysate, and all experiments were conducted with four replicates. Both telomere (TTAGGG) and scrambled (GTGAGT; control) baits were used in the BRF41 pull-down, while only telomere bait was used in the pull-down of wild-type versus *zbtb48*^*−/−*^ mutant larvae. The incubation was carried out on a rotating wheel at 4 °C for 1 h, followed by three rounds of washes with PBB buffer (150 mM NaCl, 50 mM Tris-HCl (pH 7.5), 5 mM MgCl_2_, 0.5% Igepal CA630 (Sigma), freshly supplemented with 1 mM DTT and protease inhibitors). The samples were eluted with 20 μl of 0.1 M DTT and 1X NuPAGE™ LDS Sample Buffer (Thermo Scientific), followed by boiling at 70 °C for 10 min.

### MS sample preparation

Lysates eluted from the telomere pull-down were separated on a 10% NuPAGE Bis-Tris precast gel (Thermo) at 180 V for 8 min. In-gel digestion was performed as previously described^15^. In brief, the samples were reduced with 10 mM DTT for 1 h at 56 °C, followed by alkylation with 55 mM iodoacetamide (Sigma) for 45 min in the dark. Tryptic digestion was carried out with 1 μg of MS-grade trypsin (Sigma) in 50 mM ammonium bicarbonate buffer (pH 8.0) at 37 °C overnight. Afterwards, the peptides were desalted and stored on activated C18 material (Empore) StageTips.

### MS measurement and analysis (telomere pull-down)

Measurements of telomere pull-down lysates from BRF41 and 5 dpf larvae were conducted on an Exploris 480 (Thermo) coupled online to an Easy-nLC 1200 (Thermo) on an in-house packed C18 (Dr. Maisch GmbH) column. The data were acquired via data-dependent acquisition (DDA) with a top15 method. The BRF41 data were analyzed with MaxQuant^40^ version 1.6.10.43 using the UniProt zebrafish database (39,559 entries) with standard settings except label-free quantification (LFQ), and the match between run options were activated. The 5 dpf data were analyzed with MaxQuant (2.4.2.0) using a combined Swissprot/Trembl database (47,082 entries) with the standard settings except for peptides for quantification, which was switched to unique and LFQ quantitation activated. Protein quantification was performed as previously described^41^. In brief, contaminants, reverse database hits, protein groups identified only by site, and protein groups with fewer than two peptides (with at least one classified as unique) were filtered out from the MaxQuant proteinGroups.txt file. Missing values were imputed by shifting a beta distribution, based on the LFQ intensity values, to the limit of quantitation. Volcano plots were generated from Rstudio using ggplot2^42^ and other packages. The threshold for protein enrichment was set at a fold change > |2| and a *p*-value < 0.05 (Welch’s t test).

### Orthologous comparison

Amino acid sequences and domain annotations of human ZBTB48 (P10074) and zebrafish Zbtb48 (E7FDZ5) were obtained from UniProt and aligned online^43^. The percentage of domain identity was obtained through a BLAST search.

### Zebrafish husbandry

The AB/Tübingen zebrafish strains were housed at the Institute of Molecular Biology in Mainz, and husbandry was carried out in accordance with the standards described^44^. The room and water temperature were maintained at 26–28 °C with a 14:10-h light:dark cycle. To induce embryo spawning, adult males and females were separated in a mating tank the evening prior to embryo spawning. The mating process was initiated by reuniting the fish the following morning. The embryos were collected in a 10-cm Petri dish filled with E3 medium (5 mM NaCl, 0.17 mM KCl, 0.33 mM CaCl_2_, 0.33 mM MgSO_4_) and raised in a 28.5 °C incubator until 5 days post fertilization (dpf). Afterward, they were transferred to the aquarium facility. All experiments were approved and licensed by the local authorities of Rhineland-Palatinate and were conducted in accordance with European animal welfare law. We have complied with all relevant ethical regulations for animal use.

### Creating the *zbtb48* CRISPR-Cas9 knockout zebrafish line

A pair of guide RNAs (gRNAs; Supplementary Table 1) targeting the second exon of the *zbtb48* gene (ENSDARG00000039263) were designed using CRISPRscan software (https://www.crisprscan.org). These gRNAs were cloned and inserted into the pDR274 vector and transcribed as previously described^45^. The injection mixture was prepared with 1.5 μl of each gRNA, 0.5 μl of NEB3.1, 0.5 μl of phenol red (Sigma) and 1 μl of EnGen® Spy Cas9 NLS (NEB). This mixture was injected into embryos at the one-cell stage, which were then raised to adulthood and outcrossed with wild-type fish. The strain is registered as ZDB-ALT-241104-4 at the zebrafish information network. The F1 offspring were screened for deletions in the *zbtb48* locus using PCR. These F1 mutants were outcrossed with wild-type fish for another generation to create an F2 generation before in-crossed to generate *zbtb48*^*−/−*^ mutants (F3). Additionally, the *zbtb48*^*−/−*^ mutant fish line was crossed with the wild-type *Tg(vasa:eGFP)* reporter line^34^ to label germ cells with green fluorescence.

### Genotyping of zebrafish specimens

The fish were either sacrificed on an ice bath or anesthetized with 0.02% tricaine (Sigma) for caudal fin clipping. The samples were lysed in lysis buffer (10 mM Tris-HCl (pH 8.0), 50 mM KCl, 2.5 mM MgCl2, 0.45% Igepal CA630 (Sigma), 0.45% TWEEN® 20 (Sigma), 0.01% gelatin, and 100 μg*ml^−1^ proteinase K) at 60 °C for 1 h, followed by proteinase K deactivation at 90 °C for 15 min. The PCR mixture was prepared in a 10 μl reaction volume using Taq polymerase (IMB PPCF) and the primers listed in Supplementary Table 1. The amplification was performed as follows: denaturation at 95 °C for 5 min; 5 cycles of 95 °C for 15 s, 64 °C for 30 s, and 68 °C for 30 s; 5 cycles of 95 °C for 15 s, 60 °C for 30 s, and 68 °C for 30 s; and 27 cycles of 95 °C for 15 s, 55 °C for 30 s, 68 °C for 30 s, and a final extension at 68 °C for 5 min. The PCR products were resolved by 2% agarose gel electrophoresis. The genotype distribution of offspring bred from eight *zbtb48*^*+/−*^ crosses was tested using the chi-square test. The test was performed on individual crosses and overall as a group. The outcome was determined by the X^2^ critical value of 0.05 significance level (α) and 2 degrees of freedom.

### Fish fertility

Fertility test was conducted on second-generation males aged 1 year and 6 months (*n* = 5 for wild-type and *n* = 6 for *zbtb48*^*−/−*^ mutants). The experiment was carried out in a single mating setup, using females from a single wild-type clutch that were aged 1 year 3 months. Adult males and females were separated in the mating tank the evening before embryo spawning and were reunited the following morning for mating. The eggs were collected within 30 min after laying, allowing for the removal of any defective eggs before counting the fertilized eggs around 3 h post-fertilization (hpf). The percentage of fertilized eggs (calculated as fertilized eggs divided by the total number of eggs laid) for each mating was represented in a boxplot. *P*-values were calculated using Welch’s t-test.

### Telomere restriction fragment (TRF) analysis by Southern blot

Telomere length was measured on the caudal fins of three 8-month-old males of *zbtb48*^*−/−*^ and the wild-type counterpart fish bred through group mating of *zbtb48*^*+/−*^ parents. The TRF analysis was performed as previously described^4^. In brief, the fins were lysed overnight at 50 °C using lysis buffer (Fermentas) supplemented with 1 mg*ml^−1^ Proteinase K (Sigma) and 1:100 dilution RNase A (Sigma). Genomic DNA was extracted by equilibrated phenol-chloroform (Sigma) and chloroform-isoamyl alcohol extraction (Sigma). The same amount of genomic DNA was digested with RSAI and HINFI enzymes (NEB) for 12 h at 37 °C. Samples were electrophoresed on a 0.6% agarose gel, in 0.5% TBE buffer, at 4 °C for 17 h at 110 V. Gels were then processed for Southern blotting using a 1.6 kb telomere probe, (TTAGGG)n, labeled with [α-32P]-dCTP.

### Quantitative real-time PCR

qRT‒PCR was performed on 5 dpf wild-type and *zbtb48*^*−/−*^ mutant larvae bred from parents of the respective genotype through single mating. Testes were harvested from the first generation of *zbtb48*^*−/−*^ mutants and their wild-type counterparts at 1-year-old. Each biological replicate consisted of a pool of 25 larvae or the gonad from a single individual. Samples were resuspended in 500 μl of TRIzol™ Reagent (Ambion) and sonicated by a Bioruptor Plus UCD-300I (Diagenode) at a low setting with three rounds of on for 30 s and off for 30 s, or until the samples were fully disintegrated, in a 4 °C water bath. RNA was purified using a Direct-zol RNA MicroPrep Kit (Zymo Research), and 1 μg of the extracted RNA was used for synthesizing complementary DNA (cDNA) with a First Strand cDNA Synthesis Kit (Thermo Fisher) according to the manufacturer’s instructions. qRT‒PCR was performed in technical triplicates, and each reaction consisted of a total volume of 10 μl: 5 μl of 2X Power SYBR Green PCR Master Mix (Applied Biosystems), 0.3 μl of each 10 μM primer, and 2 μl of 1:20 diluted cDNA. The primers used in this study are listed in Supplementary Table 1, and β-actin was used as a reference. The reactions were performed in a QuantStudio 5 Real-Time PCR system (Applied Biosystems) with the following amplification conditions: 50 °C for 2 min, 95 °C for 10 min, 40 cycles at 95 °C for 15 s, and 60 °C for 1 min, followed by 95 °C for 15 s, 60 °C for 1 min, and 95 °C for 15 s to determine the melting curve. The data were extracted from QuantStudioTM Design & Analysis software (Applied Biosystems) and processed with the 2 − ΔΔCt method^46^. Error bars represent the standard error of the mean (SEM), and *p*-values were calculated using two-tailed Welch’s t test.

### Tissue harvesting

To obtain total protein lysates from 5 dpf wild-type and *zbtb48*^*−/−*^ mutant larvae for the telomere pull-down experiment, approximately 200 larvae bred from parents of the respective genotype through single mating were pooled. The samples were sonicated in 2 ml of protein lysis buffer (25 mM Tris-HCl (pH 7.5), 150 mM NaCl, 1.5 mM MgCl_2_, 1% Triton™ X-100 (Sigma), 1 mM DTT, and protease inhibitors) using Bioruptor Plus UCD-300I (Diagenode) with three rounds of on for 30 s and off for 30 s, or until the samples were fully disintegrated, in a 4 °C water bath. The lysates were centrifuged at maximum speed for 30 min at 4 °C, and the supernatant was collected. The protein concentrations were measured using a Bradford assay (Bio-Rad).

For proteome analysis, 5 dpf wild-type and *zbtb48*^*−/−*^ mutant larvae were also bred from parents of the respective genotype through single mating and pooled. An additional deyolking step was performed by titrating the larvae in cold Ringer’s buffer (5 mM HEPES (pH 7.2), 116 mM NaCl, 2.9 mM KCl) supplemented with 0.3 mM PMSF and 1 mM EDTA (pH 7.0). To prepare the total protein lysates of larvae and adult testes from 10.5-month-old fish, samples were snap-frozen in liquid nitrogen and ground with micropestles before being resuspended in 50 μl of RIPA buffer. For the ovaries and testes harvested from 40 dpf juveniles, the fish were bred via single mating of *Tg(vasa:eGFP);zbtb48*^*+/−*^ parents. The fish were genotyped upon harvesting the ovaries or testes. The samples were lysed through sonication in 25 μl of RIPA buffer with the settings mentioned above. All RIPA lysis buffer was centrifuged at maximum speed for 30 min at 4 °C to collect the supernatant. The protein concentrations were measured using a Bradford assay (Bio-Rad), and 50 μg of protein was prepared for each replicate with the addition of 0.1 M DTT and 1X NuPAGE™ LDS Sample Buffer (Invitrogen), followed by boiling at 70 °C for 10 min. For 5 dpf larvae, a 10% NuPAGE Bis-Tris gel was run at 180 V for 30 min, and each lane was cut into 3 fractions.

### MS measurement and analysis (proteome)

Proteome analysis of 5 dpf larvae was conducted using an Exploris 480 (Thermo) coupled online to an in-house packed C18 (Dr. Maisch GmbH) column. For the ovaries and testes, proteomes were measured with a TimsTOF HT (Bruker) interfaced with a NanoElute2 HPLC system (Bruker). An Aurora Ultimate CSI 25 × 75 C18 UHPLC column (Ionopticks) was used at 50 °C. The peptides were eluted along a 95-min gradient from 4% to 32% acetonitrile (VWR) and measured with a DDA acquisition scheme. The measurement files were processed with MaxQuant (2.4.2.0) and a combined Swissprot/Trembl database (47,082 entries) with the standard settings except for peptides for quantification, which was switched to unique and LFQ quantitation activated. Protein analysis was performed as previously described^41^. In brief, contaminants, reverse database hits, protein groups identified only by site, and protein groups with fewer than two peptides (with at least one classified as unique) were filtered out from the MaxQuant proteinGroups.txt file. Missing values were imputed by shifting a beta distribution, based on the LFQ intensity values, to the limit of quantitation. Volcano plots were generated from Rstudio using ggplot2^42^ and other packages. The threshold for protein enrichment was set at a fold change > |2| and a *p*-value < 0.05 (Welch’s t test).

### Functional enrichment analysis

Genes were queried in the Gene Ontology database^47^ using the ClusterProfiler R package^48^. Terms found among the enriched genes were tested for over-representation with an adjusted *p*-value (false discovery rate) <0.05 (Fisher’s exact test) against terms found in the background (defined as all quantified genes in the comparison, whether enriched or not).

### Next-generation mRNA sequencing and analysis

From a single mating of *zbtb48*^*+/−*^ parents, 22 sibling larvae were picked at 5 dpf and processed individually. RNA and DNA were extracted using an AllPrep DNA/RNA/Protein Mini Kit (Qiagen) according to the manufacturer’s instructions. The larvae were sonicated in the provided RLT buffer using three rounds of Bioruptor Plus UCD-300I (Diagenode) for 30 s and off for 30 s, or until the samples were fully disintegrated, in a 4 °C water bath. Genotyping was performed on the extracted DNA, and subsequently, the RNA of four selected wild-type and *zbtb48*^*−/−*^ mutant strains was subjected to mRNA sequencing.

NGS library preparation was performed with Illumina’s TruSeq Stranded mRNA LT Sample Prep Kit following the TruSeq Stranded mRNA Reference Guide (Oct. 2017) (Document # 1000000040498v00) using ¼ of the reagents. Libraries were prepared with a starting amount of 250 ng and amplified in 10 PCR cycles. Libraries were profiled on a high-sensitivity DNA chip on a 2100 Bioanalyzer (Agilent Technologies) and quantified using the Qubit dsDNA HS Assay Kit in a Qubit 2.0 Fluorometer (Life Technologies). All 8 samples were pooled together with 20 samples from another project in equimolar ratio and sequenced on a NextSeq 500 High Output FC, SR for 1 × 75 cycles plus 2 × 8 cycles for the dual index read.

The library quality was assessed with FastQC (version 0.11.9) and FastQScreen (version 0.15.2) before alignment against the *D. rerio* genome assembly GRCz11 and its canonical gene annotations (Danio_rerio.GRCz11.100.chr.gtf) and its associated.GTF and.BED files annotations. Alignment was performed with STAR aligner^49^ version 2.7.3a (options: –runMode alignReads –outStd SAM –outSAMattributes Standard –outSJfilterReads Unique –outSAMunmapped Within –outReadsUnmapped None –outFilterMismatchNoverLmax 0.04 –outFilterMismatchNmax 999 –sjdbOverhang 75. Reads mapped to annotated features in the GTF file were counted with featureCounts^50^ version 1.6.2 using the featureCounts functionality (options: –donotsort -t exon). Coverage tracks were generated with deepTools^51^ version 3.1 (bamCoverage –binSize 1 –skipNonCoveredRegions –normalizeUsing CPM) and plotted using Gviz^52^ on an R framework. Finally, the overall quality of the reads and the alignment was assessed with MultiQC version 1.7^53^.

Further filtering and exploratory analysis were performed in an R framework including ggplot2^42^. Pairwise differential expression comparisons were performed with DESeq2^54^. Gene expression in RPKM was used to filter out individuals with a replicate average lower than 0, thus considering them as non-expressed. Differentially expressed genes (DEGs) were selected with an adjusted *p*-value (false discovery rate) of <0.01, and a threshold of at least a 1 log_*2*_-fold-change difference between conditions was applied.

### Analysis of published RNA-seq data

Metadata from RNA-seq analysis of embryo development^25–27^ and adult tissues^28^ were downloaded and processed in R.

The data and images of the scRNA measurements of 40 dpf ovaries^33^ and adult testes^29^ were obtained from the following websites: https://singlecell.broadinstitute.org/single_cell/study/SCP928/40dpf-ovary-all-cells and https://sposato.shinyapps.io/testis_shiny_app/.

### Reporting summary

Further information on research design is available in the Nature Portfolio Reporting Summary linked to this article.

## Supplementary information

Supplementary Information

Supplementary Dataset 1

Supplementary Dataset 2

Supplementary Dataset 3

Supplementary Datatset 4

Description of Additional Supplementary Files

Reporting summary

## Data Availability

The transcriptome data have been deposited in the SRA under accession number PRJNA1118296. The mass spectrometry proteomics data have been deposited to the ProteomeXchange Consortium via the PRIDE partner repository with the dataset identifier PXD052763. The data used in this paper and figures are available at Zenodo (10.5281/zenodo.14793328).
