# Supplementary material for: Zbtb48 is a regulator of Mtfp1 expression in zebrafish
Source: Commun Biol. 2025 Feb 22;8:277. doi: 10.1038/s42003-025-07666-z (PMC11846949; doi:10.1038/s42003-025-07666-z)
Supplement: Supplementary file 1 — Supplementary Information [file 42003_2025_7666_MOESM1_ESM.pdf]

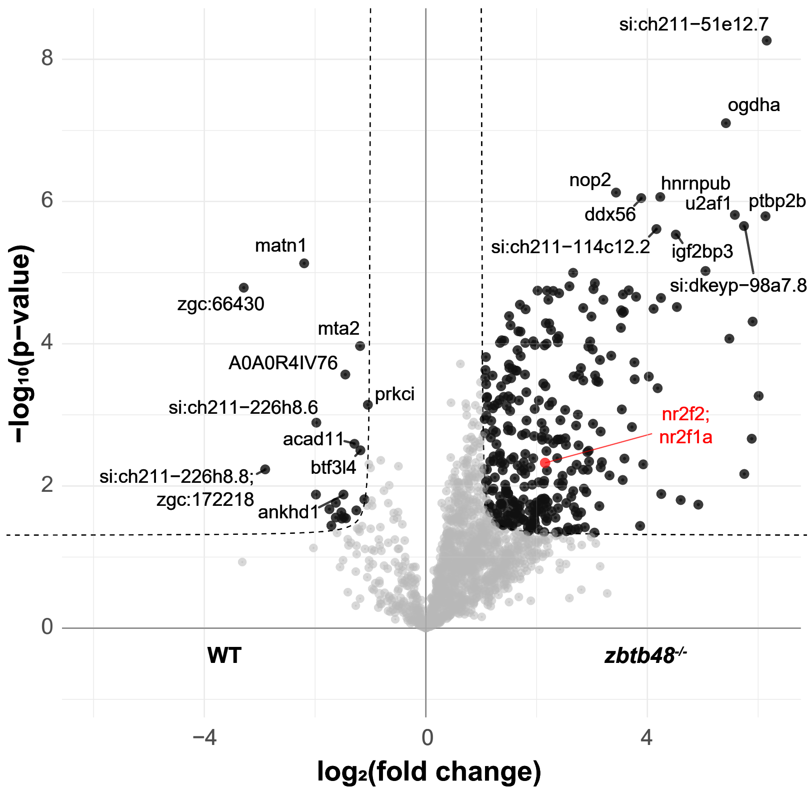

Suppl. Figure 1. Telomere pull-down between 5 dpf wild-type vs *zbtb48*$^{-/-}$ mutant larvae

Volcano plot represents the results obtained by label-free quantification of the bound proteins. The pull-down experiment was performed in quadruplicate using concatenated telomeric TTAGGG oligonucleotide as bait and protein lysates from 5 dpf wild-type and *zbtb48*$^{-/-}$ mutant samples. The results were log-transformed and plotted on the x-axis as $\log_2$(fold change) and on the y-axis as $-\log_{10}$(p-value). The protein enrichment threshold was set at a fold change > |2| and a p-value < 0.05 (Welch's t test) with c = 0.05. Enriched proteins were annotated with their gene names. The red dot highlights a protein that was previously identified in the telomere pull-down performed on BRF41 (Figure1b).

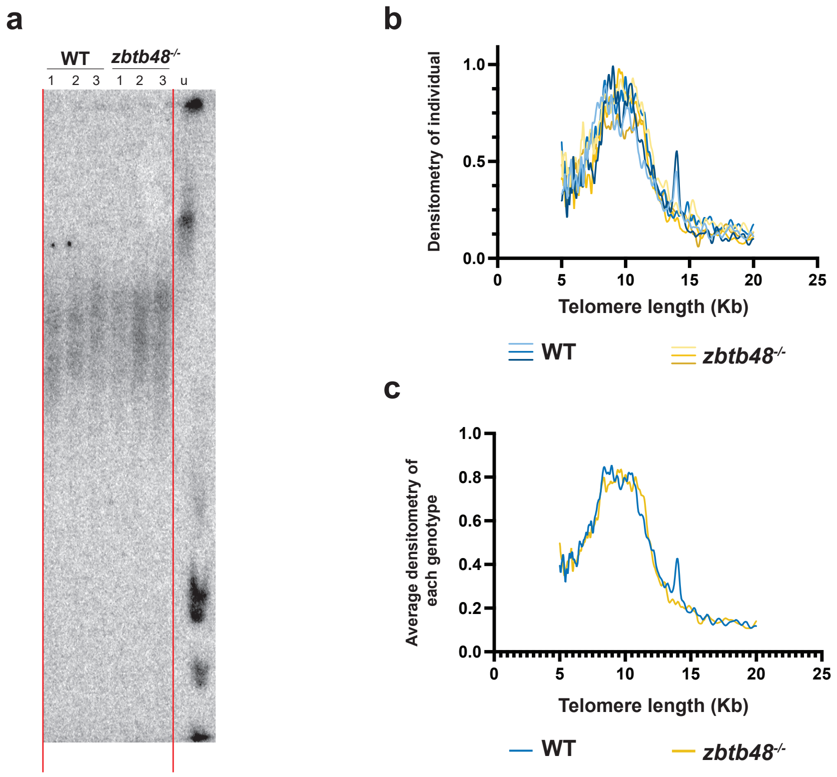

Suppl. Figure 2. Telomere restriction fragment (TRF) analysis

a, Southern blot image showing the telomere restriction fragment (TRF) of the caudal fin collected from the first generation of 8-month-old males (n=3), and 'u' represents undigested. **b,** Densitometry measurements of individual specimens. **c,** Determined averages for each respective genotype.

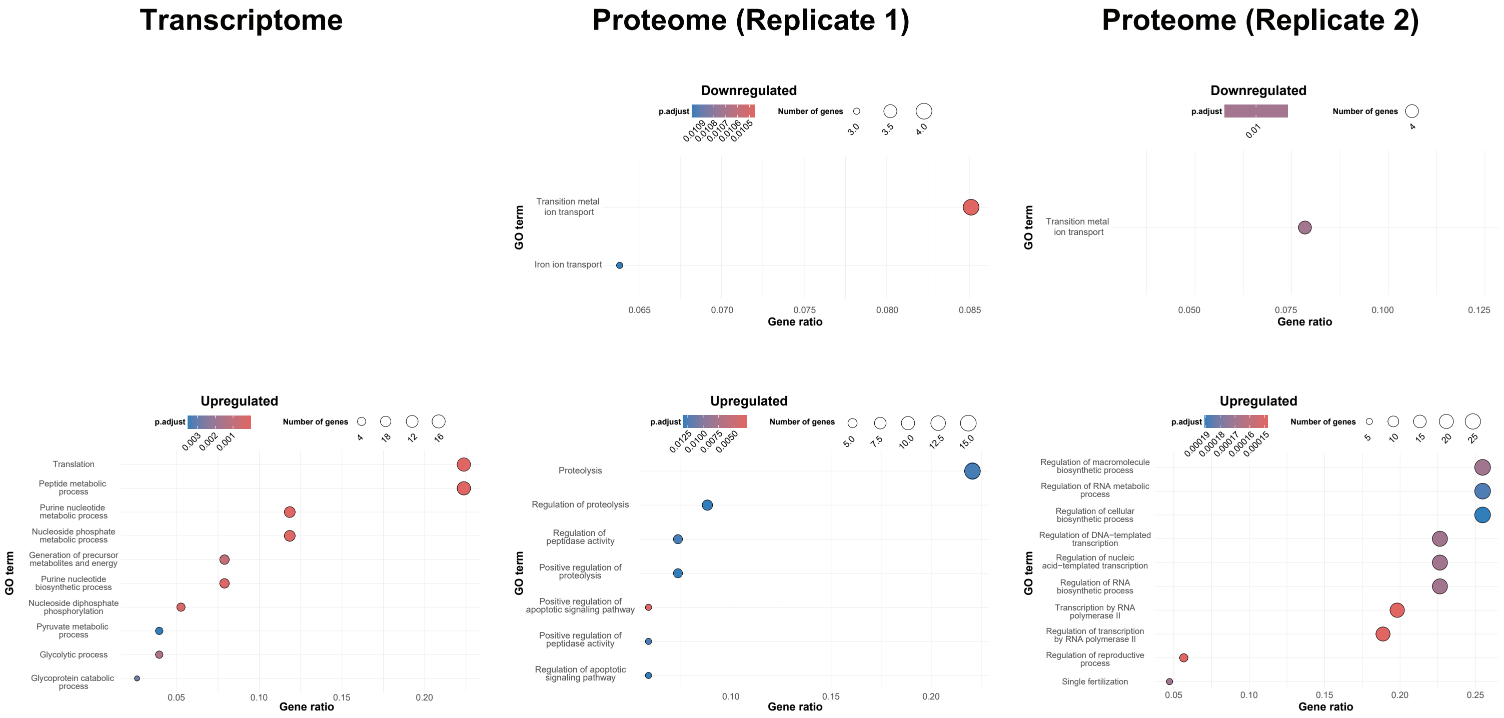

Suppl. Figure 3. GO-BP analysis on 5 dpf omics data

Gene ontology (GO) analysis conducted on 5 dpf transcriptomic (left) and both proteomic (first replicate in the middle and second replicate on the right) data showing the top 10 most significantly enriched biological processes (BP).

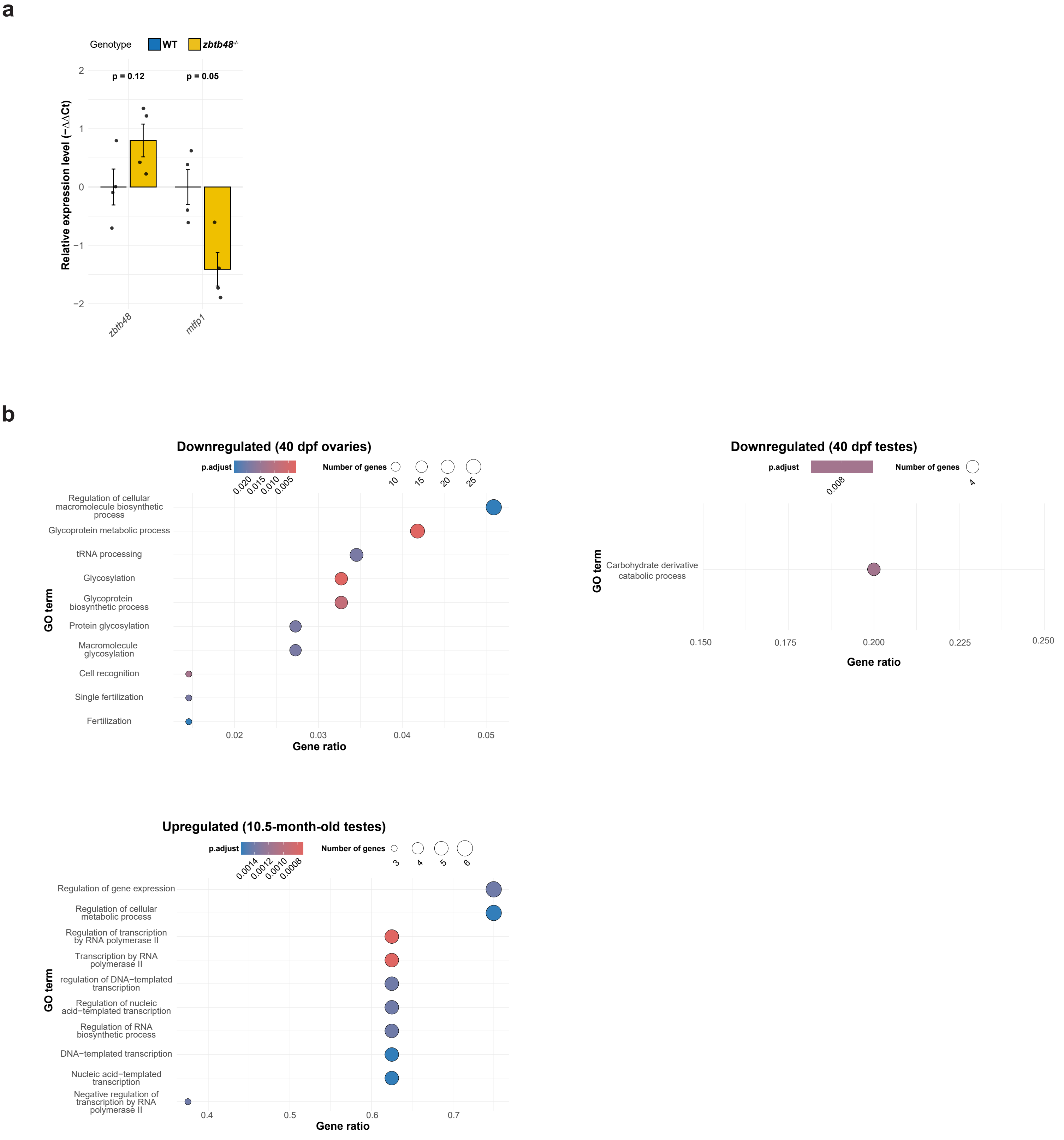

Suppl. Figure 4. GO-BP and qRT-PCR analysis on gonads

a, qRT-PCR analysis of *zbtb48* and *mtfp1* on testes of first generation wild-type and *zbtb48*$^{-/-}$ mutant males at 1 year old. The experiment was conducted in biological quadruplicate (n=4 males) with technical triplicates each. Error bars represent the standard error of the mean (SEM), and p-values were calculated using two-tailed Welch's t test. **b,** Gene ontology (GO) analysis conducted on proteomic data from the ovaries of 40 dpf (top left), testes of 40 dpf (top right) and testes of 10.5-month-old samples (bottom left), showing the top 10 most significantly enriched biological processes (BP).

Suppl. Table 1: Primers used in this study.

**Primer name**	**Sequence (5'-3')**
zbtb48 gRNA1 Top	tagg CAGGCCAGGACATTGCGAT
zbtb48 gRNA1 Btm	aaac ATCGCAATGTCCTGGCCTG
zbtb48 gRNA2 Top	agg CATCGGGACACTCTAAAGGG
zbtb48 gRNA2 Btm	aaac CCCTTTAGAGTGTCCCGATG
zbtb48 genotyping forward	ATGGAGAAACTTGCAGAA
zbtb48 genotyping reverse	CCCCAGAACATCAGGTTAATGGCG GGTTTTGATGGATCACCTTCA
β- actin- FW	CCTCACTTTGAGCTCCTCCAC
β- actin- RW	GACCCACGATGGATGGGAAG
zbtb48- FW	ACATTTCGCACTCAAGCCAG
zbtb48- RW	GTGTGGTCGACCTTCTTGGT
mtfp1- FW	CTGGGTCAGATTCCTGGGTT
mtfp1- RW	GCCTTTATCGATGGCGTCTG
zgc:153284- FW	TTCCTGGTCCTTGAGTTGGC
zgc:153284- RW	ACCCTGATGTTGCTTTACCTCT
akr7a3- FW	CACCGCGCTGATGTACAATG
akr7a3- RW	GGTCGCGATTCGAACTGTTT
atox1- FW	ACACTGATGTCCTTCTGGAAACA
atox1- RW	GCAGCATGATTCTGACCTTATTGT
col28a2a- FW	TGAATGTGGACACGAAGCCT
col28a2a- RW	CCAGGGGAGCCGATTCTTTTA
hdac10- FW	GGGGACCCAGAAGGTGAAAT
hdac10- RW	TTTTTCCTGCAGCCAAAGGC
pxmp2- FW	TGTTCGAGACCCCTCTTTGC
pxmp2- RW	AGCAGACAGAATACCACTCGT
vwa5a- FW	CTCCGATGGCTCGCTGTTTA
vwa5a- RW	GTGCAGACTTTGCTTCACGG
ppp3cb- FW	GAAAGGACGACTAAAAGCGGTG
ppp3cb- RW	CAAACACCTCTCGCATGCTC
