# Supplementary material for: Zbtb48 is a regulator of Mtfp1 expression in zebrafish
Source: Commun Biol. 2025 Feb 22;8:277. doi: 10.1038/s42003-025-07666-z (PMC11846949; doi:10.1038/s42003-025-07666-z)
Supplement: Supplementary file 6 — Description of Additional Supplementary Files [file 42003_2025_7666_MOESM6_ESM.pdf]

Description of additional supplementary file

File name: Supplementary Data 1
Description: A list of proteins quantified from telomere pull-down assays by mass spectrometr y using label-free quantification.
'BRF41- Quantified proteins' tab: Telomere pull-down experiment conducted on nuclear lysates from the BRF41 zebrafish fin fibroblast cell line, comparing the proteins enriched from telomere sequ ence to a scrambled control sequence.
'5dpf- Quantified proteins' tab: Telomere pull-down experiment comparing lysates from wild-type and *zbtb48*$^{-/-}$ mutant larvae at 5 dpf.

File name**:** Supplementary Data 2
Description: Transcriptomic (RNA-seq) analysis of 5 dpf lavae.
'DESeq2' & 'Count_RPKM' tab: A list of transcripts quantified from *zbtb48*$^{-/-}$ mutants (n=4) and their wild-type siblings (n=4) at 5 dpf. GO-BP' tab: A complete list of GO-BP terms that were found significantly (p < 0.05) dysregulated.

File name: Supplementary Data 3
Description: Proteome analysis of 5 dpf lavae (Replicate 1 & 2).
'Quantified proteins' tab: A list of proteins quantified by mass spectrometry using label-free quantification, from zbtb48-/- mutants and their wild-type counterparts at 5 dpf. The experiment was performed in quadruplicate, with each replicate consisting of a pool of 25 larvae.
'GO-BP' tab: A complete list of GO-BP terms that were found significantly (p < 0.05) dysregula ted.
'Dysregulated genes' tab: Gene descriptions of those commonly dysregulated in the omics analyses a t 5 dpf.

File name: Supplementary Data 4
Description: Proteome analysis of adult fish gonads (ovaries from 40 dpf fish, testes from 40 dpf and 10.5-month-old fish).
'40dpf Ovaries- Quantified proteins' tab: A list of proteins quantified by mass spectrometry using label-free quantification on ovaries obtained from zbtb48-/- mutants (n=3) and their wild-type siblings (n=3) at 40 dpf.
'40dpf Testes-Quantified proteins' tab: A list of proteins quantified by mass spectrometry using label-free quantification on testes obtained from zbtb48-/- mutants (n=4) and their wild-type siblings (n=4) at 40 dpf.
'10.5mpf Testes-Quantified proteins' tab: A list of proteins quantified by mass spectrometry using label-free quantification on testes obtained from second generation of zbtb48-/- mutants (n=4) and their wild-type counterpart (n=4) at 10.5-month-old.
'GO-BP' tab: A complete list of GO-BP terms that were found significantly (p < 0.05) dysregula ted.
